# Androgen receptor expression predicts breast cancer survival: the role of genetic and epigenetic events

**DOI:** 10.1186/1471-2407-12-132

**Published:** 2012-04-02

**Authors:** Kate M Peters, Stacey L Edwards, Shalima S Nair, Juliet D French, Peter J Bailey, Kathryn Salkield, Sandra Stein, Sarah Wagner, Glenn D Francis, Susan J Clark, Melissa A Brown

**Affiliations:** 1 School of Chemistry and Molecular Biosciences, The University of Queensland, St. Lucia 4072 Queensland, Australia; 2 Epigenetics Group, Garvan Institute of Medical Research, Sydney 2010, NSW, Australia; 3 Department of Pathology, Princess Alexandra Hospital, Queensland Woolloongabba, 4102 Queensland, Australia

**Keywords:** Androgen receptor, Prognostic biomarker, Breast cancer, Gene regulation, Promoter methylation, Regulatory mutation, MiRNA

## Abstract

**Background:**

Breast cancer outcome, including response to therapy, risk of metastasis and survival, is difficult to predict using currently available methods, highlighting the urgent need for more informative biomarkers. Androgen receptor (AR) has been implicated in breast carcinogenesis however its potential to be an informative biomarker has yet to be fully explored. In this study, AR protein levels were determined in a cohort of 73 Grade III invasive breast ductal adenocarcinomas.

**Methods:**

The levels of Androgen receptor protein in a cohort of breast tumour samples was determined by immunohistochemistry and the results were compared with clinical characteristics, including survival. The role of defects in the regulation of Androgen receptor gene expression were examined by mutation and methylation screening of the 5' end of the gene, reporter assays of the 5' and 3' end of the AR gene, and searching for miRNAs that may regulate AR gene expression.

**Results:**

AR was expressed in 56% of tumours and expression was significantly inversely associated with 10-year survival (P = 0.004). An investigation into the mechanisms responsible for the loss of AR expression revealed that hypermethylation of the *AR *promoter is associated with loss of AR expression in breast cancer cells but not in primary breast tumours. In AR negative breast tumours, mutation screening identified the same mutation (T105A) in the 5'UTR of two AR negative breast cancer patients but not reported in the normal human population. Reporter assay analysis of this mutation however found no evidence for a negative impact on *AR *5'UTR activity. The role of miR-124 in regulating AR expression was also investigated, however no evidence for this was found.

**Conclusion:**

This study highlights the potential for AR expression to be an informative biomarker for breast cancer survival and sets the scene for a more comprehensive investigation of the molecular basis of this phenomenon.

## Background

Breast cancer is a heterogeneous disease comprising tumour subtypes associated with variable clinical characteristics [1]. Variables including tumour size, histological subtype and grade, lymph node status and the expression of estrogen receptor alpha (ERα), progesterone receptor (PR) and human epidermal growth factor receptor 2 (HER2) currently assist routine clinical management [2]. However, these factors are limited in their ability to predict individual survival and response to therapy [2]. This is particularly apparent for patients with advanced breast cancer, which is characterised by high histological grade and the presence of lymph node metastases, and has an aggressive clinical course and generally a poor prognosis [2]. Identifying new prognostic biomarkers and the molecular mechanisms underlying breast cancer progression are paramount for improving the clinical management of these patients and developing improved therapeutic strategies.

Androgen receptor (AR) is a member of the nuclear receptor superfamily and is known to be involved in a complex network of signalling pathways that collectively regulate cell proliferation [3,4]. Expressed in the normal human mammary gland, where it predominantly localises to the inner layer of epithelial cells lining acini and intralobular ducts [5], the role of AR in normal mammary epithelial biology is unknown. AR has been implicated in breast tumourigenesis, however delineating its precise function has proven difficult with AR-mediated androgenic effects shown to both stimulate and inhibit growth of breast cancer cells [6,7]. The significance of AR in human breast cancer is further emphasized by the recent finding that it can be targeted in estrogen receptor negative breast tumours [8]. Loss of AR expression is associated with early onset, high nuclear grade and negative ER, PR and HER2 expression status in breast tumours [9,10]. However, the mechanisms responsible for this loss of AR expression in breast carcinogenesis remain unclear.

**Figure 1:**
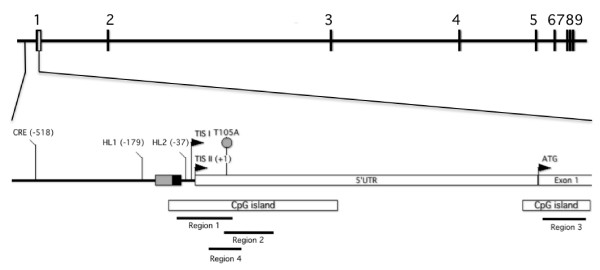
**Schematic diagram of the human *AR *gene**. The relative positions of the two transcription initiation sites (TIS I and II) and functionally known motifs; CpG islands, cAMP responsive element (CRE), helix-loop-helix-like (HL) motifs, a palindromic homopurine repeat (grey box) and GC-box (black box) which contains an SP1 binding site, are indicated (GenBank Accession No. NG009014.1). A grey circle denotes the T105A alteration identified in two primary breast tumours in the present study. DNA methylation was assessed in Regions 1-4, as denoted by black lines. *UTR*, untranslated region. *ATG*, translation start site.

The *AR *gene comprises 9 exons spanning 180.25 kilobases located on chromosome Xq12. Functional analyses have identified two independently regulated transcription initiation sites (TIS), *AR*-TIS I (-12/-11/-10) and *AR*-TIS II (-1/+1) (Figure 1) [11]. Transcriptional initiation from *AR*-TIS I is dependent on sequences located between positions -17 and +45 and initiation from *AR*-TIS II facilitated by a palindromic homopurine repeat and SP1 binding to a GC-box [12,13]. Additional putative *cis*-acting elements include HL (helix-loop-helix-like) motifs 1 and 2 [14] and a cAMP responsive element [15]. Two CpG islands (CGI) are also located in the *AR *promoter and extend into Exon 1. Hypermethylation of these CGI have been shown to silence *AR *transcription in prostate cancer cells and primary tumours [16]. Genetic alterations in the promoter and 5'untranslated regions (UTR) of the *AR *gene have been also observed in prostate cancer cell lines, xenografts [17] and in two prostate cancer patients [18,19]. In breast cancer, the role of regulatory defects in the AR gene are yet to be fully elucidated.

In this study, we show that loss of AR expression is significantly associated with poor 10 year survival outcome in Grade III invasive breast ductal adenocarcinomas. We then evaluated potential regulatory mechanisms that may account for the loss of AR expression. For the first time we show that DNA hypermethylation in the *AR *promoter is associated with loss of AR expression in breast cancer cells, although this is not the case in our cohort of tumours from patients with Stage III breast cancer. We subsequently assessed whether somatic mutations in AR regulatory regions or miRNAs bioinformatically predicted to target the human *AR *3'UTR might contribute to the observed changes in AR expression.

## Results and Discussion

### Low AR protein levels are associated with poor 10-year survival in patients with Stage III breast cancer

**Figure 2:**
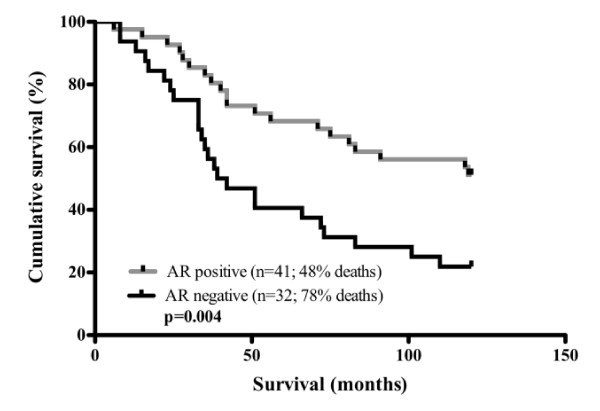
**Impact of Androgen Receptor expression on breast cancer survival**. Kaplan-Meier estimates of 10 year survival of patients by AR expression for primary tumours. n denotes the number of patient samples.

To assess the prognostic value of AR expression in breast cancer patients, IHC analysis was performed in a cohort of 73 Grade III lymph node positive ductal adenocarcinomas from patients with Stage III disease. Patient and tumour characteristics are summarised in Table 1. The patients ranged in age from 30 to 94 years (mean, 54 years); with the majority of patients (97%) aged over 35 years. AR expression was detected in 56% (n = 41) of primary breast tumours. Positive expression of ER, PR and HER2 was also observed in 55.5% (n = 40), 40% (n = 29) and 21.7% (n = 15) of breast tumours, respectively. In AR-negative tumours, the majority (72%, n = 23; 87%, n = 27; 86.6%, n = 26) were also ER, PR and HER2 negative, respectively. The authors acknowledge the potential limitations of TMA analysis, given the inherent heterogeneity of tumour samples, but note the evidence that there is a high concordance between TMA cores and whole sections [20]. In addition the impact was further minimized by analysing at least two cores from each tumour, in accordance with the correlation nomograms developed by Karlsson et al., 2009 [21]. AR expression was a significant prognostic factor for overall patient survival (P = 0.004) (Figure 2). The 10-year survival of patients with AR positive tumours was 52% versus 22% for patients with AR negative tumours. This finding is consistent with previous studies in a diversity of breast cancer patient populations wherein a significant association between AR expression and age at diagnosis, nuclear grade, recurrence-free survival was observed [9,10,22-24].

**Table 1:** Patient and tumour characteristics*

Factor	AR negative n = 32 (%)	AR positiven = 41 (%)
Age (years)		

≤35	1 (3.0)	1 (2.4)

≥35	31 (97.0)	40 (97.6)

Estrogen receptor		

Negative	23 (72.0)	9 (22.5)

Positive	9 (28.0)	31 (77.5)

Progesterone receptor		

Negative	27 (87.0)	16 (39.0)

Positive	4 (13.0)	25 (61.0)

HER2		

Negative	18 (85.7)	25 (75.8)

Positive	3 (14.3)	8 (24.2)

Triple negative^a^		

No	6 (28.6)	30 (90.9)

Yes	15 (71.4)	3 (9.1)

*All patients were diagnosed with Stage III disease, as defined in the Materials and Methods section of the manuscript

^a^Triple negative breast cancer represents tumours displaying negative expression for estrogen receptor, progesterone receptor and HER2 by IHC. AR, androgen receptor; HER2, human epidermal growth factor receptor type 2.

### DNA methylation of the *AR *promoter is associated with low *AR *mRNA levels in breast cancer cell lines

**Figure 3:**
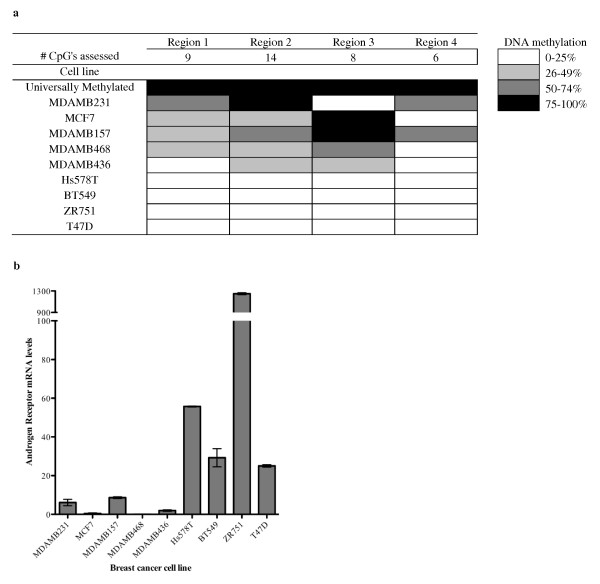
**DNA methylation of the *AR *gene is associated with loss of AR protein expression in breast cancer cells**. **(a**) DNA methylation status of breast cancer cell lines. Three regions of *AR *were assessed by MS-HRM, overlapping Regions 1 and 2 in the *AR *minimal promoter and Region 3 at the translational start site (refer to Figure 1). Region 4 was assessed by Sequenom MassARRAY. Data represent the average of two independent experiments. (**b**) AR mRNA expression in breast cancer cell lines was assessed by qPCR. Expression is shown relative to β-actin and bars represent the mean ± standard deviation of two independent experiments.

To investigate potential mechanisms responsible for the loss of AR expression, we evaluated the methylation status of the *AR *promoter region and *AR *expression levels in breast cancer cell lines. DNA methylation was determined by MS-HRM analysis of bisulfite treated DNA in three regions; Regions 1 and 2 correspond to the CpG island in the *AR *minimal promoter [14] and Region 3 corresponds to the CpG island at the translational start site (Figure 1). DNA methylation was also assessed in a further region, Region 4, by Sequenom MassARRAY (Figure 1). Cell lines, MDAMB231, MCF7, MDAMB157, MDAMB468 and MDAMB436 all showed between 25-100% methylation in at least 2 of regions analysed (Figure 3a). Notably, methylation of the *AR *promoter region was associated with the level of *AR *mRNA (Figure 3a and 3b). A similar association has been observed in prostate cancer, where treatment of prostate cancer cell lines that display *AR *hypermethylation with the demethylating agent 5-aza-2'-deoxycytidine induces the re-expression and function of AR [25].

### *AR *promoter methylation is not associated with low AR protein levels in primary breast tumours

**Figure 4:**
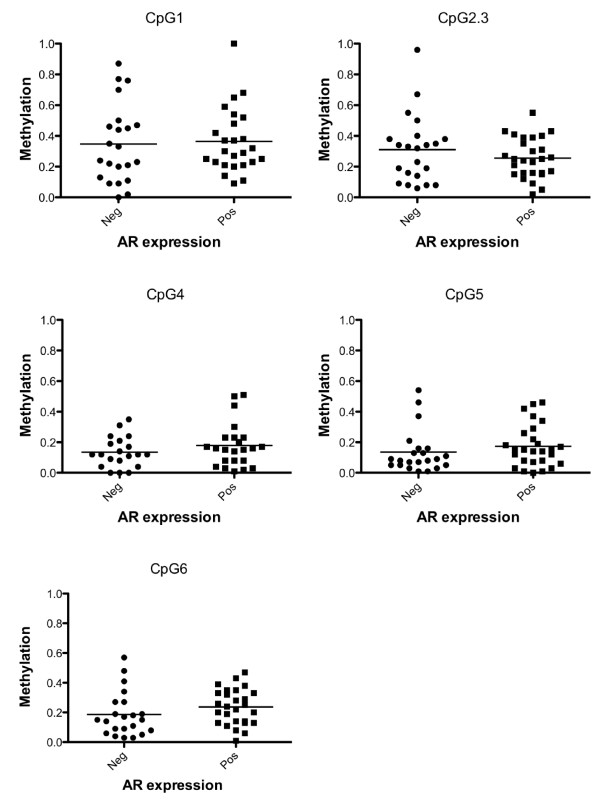
**DNA methylation of an 115 bp region in the *AR *promoter is not associated with loss of AR protein expression in a selective group of primary breast tumours**. Methylation of six CpG dinucleotides in the *AR *promoter of AR positive (Pos) and negative (Neg) Grade III breast tumours as determined by Sequenom MassARRAY (Region 4, Figure 1). Due to cleavage patterns, the average methylation was determined for CpG dinucleotides 2 and 3. Each dot represents an individual patient. A methylation value of 1.0 indicates a fully methylated amplicon, while a value of 0.0 indicates a fully unmethylated amplicon. Horizontal lines represent average cohort methylation.

To examine whether *AR *promoter methylation is also associated with loss of AR protein levels in breast tumours, the DNA methylation status of Region 4, which contains six CpG dinucleotides, was assessed by Sequenom MassARRAY. Sequenom was chosen for this analysis as it can analyse methylation at each individual CpG dinucleotide, reliably detecting methylation as low as 5%, in a high-throughput manner [26]. Primer design constraints meant that Region 4 of the *AR *promoter (Figure 1) was selected for analysis, with DNA methylation of overlapping Regions 1 and 2 associated with *AR *mRNA levels in breast cancer cell lines. DNA methylation was observed in breast cancer patients at each of the six CpGs (Figure 4). However, with the exception of CpG's 1-3, at which methylation in most tumours was greater than 30%, for the most part only low level methylation (< 30%) was observed. Furthermore, no significant association was observed in the average methylation between AR negative and AR positive primary breast tumours in our cohort at any of the six CpG dinucleotides examined.

There are several plausible explanations for the lack of association between promoter methylation and expression in primary breast tumours. There is the possibility that a region outside that examined shows expression associated methylation and indeed, there was an incomplete association between Region 4 methylation and AR mRNA levels in breast cancer cells. Additionally, another mechanism such as somatic mutation of these regions, or aberrant targeting by a miRNA, may be involved.

### Identification of somatic mutations in the *AR *5'UTR in AR negative breast tumours

Somatic mutation of regulatory regions of the *AR *gene is another potential mechanism responsible for reduced AR expression in breast tumours. To address this possibility, we sequenced the 5' regulatory region of *AR *(-659 to +280) in breast cancer cell lines. Our results revealed no sequence variations in MDAMB157, MDAMB231, MDAMB436, MDAMB468, MCF7, T47D, ZR75-1, Hs578T and BT549 cells. We also sequenced the *AR *promoter region from -6 to +133 in 32 primary breast tumours. Amplification of the *AR *promoter region from -165 to -7, which corresponds to the homopurine repeat and GC box (Figure 1), revealed a mutation (mRNA pos 105, T > A, Figure 1), mapping to the *AR *5'UTR, in two patients. This sequence variation does not correspond to any known SNPs (GRCh37 reference primary assembly, http://www.ncbi.nlm.nih.gov/sites/entrez?db=snp) or, to our knowledge, any previously reported *AR *variants.

To investigate the potential significance of the *AR *5'UTR T105A variant, we performed bioinformatics analysis on the wild-type and variant sequence. In the wild-type sequence the T position is invariant in mammals and is a component of the binding site for RNApolII (based on ChIP-seq data) and the predicted and conserved binding site for several transcription factors, including RUNX1, En1 and Pax6. Based on the currently available ChIP-seq data however, there is currently no evidence that these transcription factors bind to this sequence in vivo. The substitution from T to A results in an abolishment of these predicted sites and the creation of predicted and conserved binding sites for NHLH1 (data not shown).

**Figure 5:**
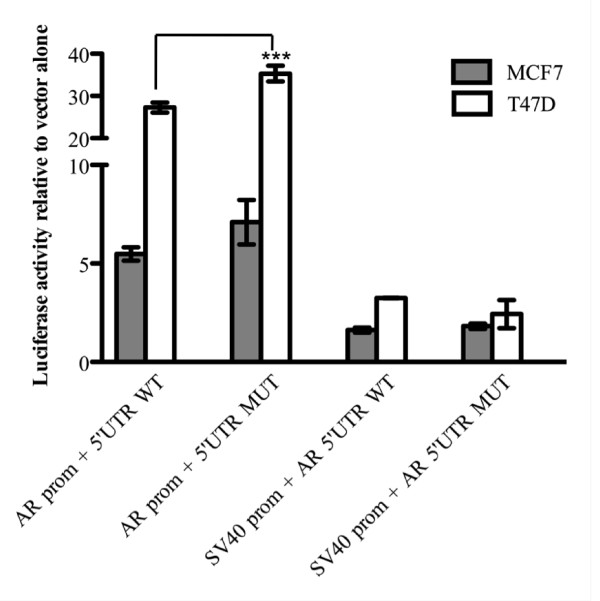
**Impact of the *AR *T105A 5'UTR variant**. Luciferase reporter activities of pGL3 basic with the *AR *promoter (*AR *prom) and pGL3 promoter vector which contains the SV40 promoter (SV40 prom) together with either the wild-type (WT) or T105A mutant (MT) *AR *5'UTR sequence in MCF7 and T47D cells. Data is shown relative to the respective empty vector ± the standard error of the mean (SEM) and was generated from three independent experiments. ***P = 0.0001.

To experimentally address the effect of this variant on 5'UTR activity, we fused the 5'UTR upstream of the firefly cDNA and downstream of either the *AR *or the SV40 promoter, in pGL3-basic and pGL3-promoter vectors, respectively. The *AR *5'UTR T105A sequence variant did not have a negative impact on SV40 or *AR *driven reporter activity in either MCF7 or T47D cells (Figure 5). Instead, the *AR *5'UTR T105A sequence variant actually increased *AR *reporter driven activity in T47D cells (P = 0.0001) (Figure 5).

Somatic mutation analysis of AR negative breast tumours identified the same alteration (chrX:66680703, mRNA pos 105, T > A) in the *AR *5'UTR of two breast tumours. Examination of the functional importance of this sequence variation however revealed that there was no negative impact on the activity of the *AR *5'UTR in MCF7 or T47D cells. This suggests that this mutation is unlikely to negatively affect the regulation of AR expression. However, particularly given the *AR *5'UTR T105A sequence variant significantly increased *AR *reporter driven activity in T47D cells, more complex studies, such as analysing the consequence of this mutation in the context of the entire *AR *gene and AR protein expression, will be required to firmly establish this. Although further analysis of our tumours was constrained by the availability and nature of the FFPE tumour material, it is plausible that mutations that effect AR expression exist outside the promoter region examined, particularly in regions upstream of the *AR *5'UTR and in the *AR *3'UTR, which are reported to contain putative regulatory elements involved in controlling mRNA stability [27]. A recent epidemiological meta-analysis of the *AR *gene concluded that common polymorphisms in the *AR *gene are not associated with breast cancer risk among Caucasian women [19]. However, the functional significance of these variants, the AR expression status and survival outcome of these patients was not considered in this study.

### miR-124 does not regulate the *AR *3'UTR in breast cancer cells

**Figure 6:**
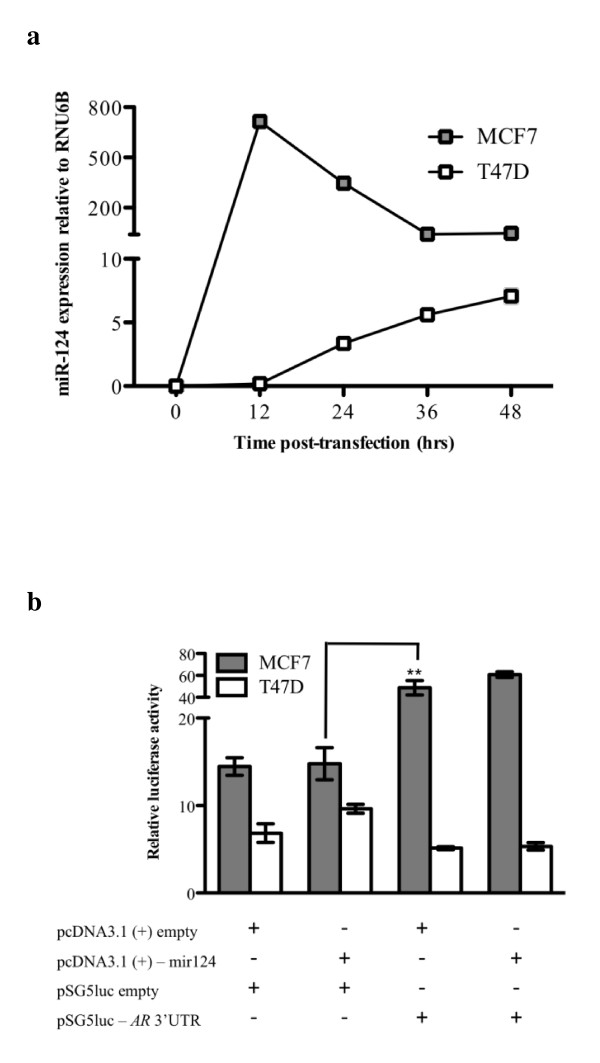
**miR-124 does not target the *AR *3'UTR in breast cancer cells**. **(a**) miR-124 expression in MCF7 and T47D cells was assessed by qPCR following transfection with pcDNA 3.1(+)-mir-124. Expression is shown relative to RNU6B and dots represent the mean ± standard deviation of two independent experiments. (**b**) Luciferase reporter activities relative to the *Renilla *internal control ± the standard error of the mean (SEM) are shown. Data were generated from three independent experiments. **P = 0.007.

MiRNAs are small non-coding RNAs of ~20nt in length that are capable of modulating gene expression post-transcriptionally. Many have been shown to act as either oncogenes or tumour suppressor genes that are crucial to the development of breast cancer metastasis and survival outcome [28]. To address the possibility that altered expression of AR is mediated by differential expression of miRNAs, we screened the *AR *3'UTR for potential miRNA target sites. Bioinformatic analysis revealed that miR-124 was the only miRNA predicted to target the human *AR *3'UTR using miRanda and TargetScan. To examine whether miR-124 regulates the expression of the *AR *transcript, we used a reporter gene assay. MCF7 and T47D cells were transfected with pcDNA 3.1(+)-mir-124 vector and expression of miR-124 verified. Cells transfected with pcDNA 3.1(+)-mir-124 expressed high levels of mature miR-124 at 12, 24, 39 and 48 hr time intervals post-transfection, whereas no endogenous expression was detected in control-transfected cells (Figure 6a). For luciferase assays, we co-transfected MCF7 and T47D cells with pcDNA 3.1(+)-mir-124 vector with the pSG5-*AR *3'UTR vector. The introduction of the *AR *3'UTR into pSG5 luc significantly increased reporter activity in MCF7 (P = 0.007) but not in T47D cells (Figure 6b). miR-124 overexpression did not alter luciferase activity of the *AR *3'UTR construct in any of the cell lines examined (Figure 6b). These results suggest that miR-124 is unlikely to regulate AR expression in these cells.

MiR-124 was the only miRNA predicted by two commonly used algorithms to target the *AR *3'UTR. However, over-expression of miR-124 did not regulate AR expression in MCF7 or T47D cells, which otherwise display no endogenous expression of this miRNA. This suggests that either miR-124 is unable to regulate AR in this particular experimental system, or that the prediction not correct. There is certainly evidence suggesting that although a plethora of targets are predicted for miRNAs, that many of these are false positives [29]. There have been reports of other miRNAs regulating the expression of AR, including miR-488 [30]. It would be of interest to determine whether there is an association between the levels of these miRNAs and AR in Stage III breast cancer, and whether these miRNAs have the potential to be informative biomarkers or therapeutic targets for this disease. Interestingly, the inclusion of the *AR *3'UTR significantly increased reporter activity in MCF7 but not in T47D cells suggesting the potential cell specific importance of this region in the post-transcriptional regulation in breast cancer cells.

## Conclusions

In this paper we show that AR expression is significantly associated with 10-year survival outcome in patients with Stage III breast cancer. To predict and potentially address the poor survival outcome of patients with AR negative breast tumours it is important to understand the mechanism underlying the reduced AR expression. Here we demonstrate for the first time that hypermethylation of sections of the *AR *5' regulatory region is associated with loss of AR expression in breast cancer cell lines, although not in a small set primary tumours. We describe a new somatic mutation in the AR 5'UTR which is found in two independent tumours and is not a normal polymorphism. This study highlights the potential for AR expression to be an informative prognostic biomarker for breast cancer survival and sets the scene for a more comprehensive investigation of the molecular basis of this phenomenon.

## Methods

### Breast cancer cell lines

Breast cancer cell lines MDAMB157 (ER-PR-), MDAMB231 (ER-PR-HER2-), MDAMB436 (ER-PR-), MDAMB468 (ER-PR-), MCF7 (ER + PR + HER2-), T47D (ER + PR + HER2-), ZR75-1 (ER + PR-), Hs578T (ER-PR-HER2-), and BT549 (ER-PR-) were obtained from American Type Culture Collection (ATCC) and cultured according to the manufacturer's recommendations. Hormone receptor status sourced from [31].

### Clinical samples

Primary breast tumours were sourced from the Princess Alexandra Hospital (Brisbane, Australia) following procedures endorsed by both The National Ethics Application process of the National Health and Medical Research Council of Australia (http://www.neaf.gov.au) and The University of Queensland Human Ethics committee. Tumour tissues were formalin fixed and paraffin embedded (FFPE), sectioned and stained using hematoxylin and eosin (H&E), and confirmed by a qualified pathologist (G.D.F.) as invasive Grade III ductal adenocarcinomas. After the exclusion of patients for whom there was no follow-up data, non-breast cancer associated deaths and unreadable IHC expression, 73 patients were available for further analysis. None of the patients had received preoperative radiochemotherapy. The characteristics of the patients and tumours is shown in Table 1, with the following definitions of breast cancer stage III:

### Stage IIIA

• no tumor is found in the breast. Cancer is found in axillary lymph nodes that are attached to each other or to other structures, or cancer may be found in lymph nodes near the breastbone; or

• the tumor is 2 centimeters or smaller. Cancer has spread to axillary lymph nodes that are attached to each other or to other structures, or cancer may have spread to lymph nodes near the breastbone; or

• the tumor is larger than 2 centimeters but not larger than 5 centimeters. Cancer has spread to axillary lymph nodes that are attached to each other or to other structures, or cancer may have spread to lymph nodes near the breastbone; or

• the tumor is larger than 5 centimeters. Cancer has spread to axillary lymph nodes that may be attached to each other or to other structures, or cancer may have spread to lymph nodes near the breastbone.

### Stage IIIB

In stage IIIB, the tumor may be any size and cancer:

• has spread to the chest wall and/or the skin of the breast; and

• may have spread to axillary lymph nodes that may be attached to each other or to other structures, or cancer may have spread to lymph nodes near the breastbone.

• Cancer that has spread to the skin of the breast is inflammatory breast cancer. See the section on Inflammatory Breast Cancer for more information.

### Stage IIIC

In stage IIIC, there may be no sign of cancer in the breast or the tumor may be any size and may have spread to the chest wall and/or the skin of the breast. Also, cancer:

• has spread to lymph nodes above or below the collarbone; and

• may have spread to axillary lymph nodes or to lymph nodes near the breastbone.

• Cancer that has spread to the skin of the breast is inflammatory breast cancer. See the section on Inflammatory Breast Cancer for more information.

Stage IIIC breast cancer is divided into operable and inoperable stage IIIC.

In operable stage IIIC, the cancer:

• is found in ten or more axillary lymph nodes; or

• is found in lymph nodes below the collarbone; or

• is found in axillary lymph nodes and in lymph nodes near the breastbone.

• In inoperable stage IIIC breast cancer, the cancer has spread to the lymph nodes above the collarbone.

### Tissue microarray blocks and immunohistochemical staining

Tumour-rich tissue from each biopsy was distinguished from surrounding normal tissue in H&E-stained sections by a qualified pathologist (G.D.F). Tissue microarrays were constructed in duplicate from tumour-rich tissue cores (1 mm × 0.6 mm) using an automated tissue microarray (TMA) instrument (ATA-27; Beecher Instruments). 4 μM sections of the TMA blocks were used for immunohistochemical (IHC) analysis. Sections were transferred on to glass slides, deparaffinised and immunostained using anti-ER (SP1), anti-PR, (SP2), anti-HER2/neu (4B5) (Ventana Medical Systems, pre-diluted) or anti-AR (Biocare Medical, 1:50 dilution) antibodies, and counterstained with 3,3'-Diaminobenzidine (DAB) and hematoxylin. Staining was performed with the BenchMark automated slide stainer (Ventana) using the iVIEW DAB detection kit with additional Avidin and Biotin Blockers according to the manufacturer's instructions. Analysis of stained sections was performed by a qualified pathologist (G.D.F) and the presence of tumour tissue confirmed by examining the counterstain. Expression was scored as positive when visible staining ≥ 1% was observed in the nucleus for ER, PR and AR. For HER2 IHC was semiquantitatively evaluated with a score of 3+ regarded as positive, 2+ as equivocal, and 1+ or 0 as negative. In instances of an equivocal evaluation, silver-enhanced in situ hybridisation was performed as previously described [32].

### DNA methylation analysis

Genomic DNA was isolated from cell lines using the NucleoSpin Tissue kit (MachereyNagel) according to the manufacturer's instructions. For each human tumour sample, four FFPE tumour-rich tissue cores (1 mm × 0.6 mm) were crushed and digested with proteinase K at 55°C for 2 days and treated with 20 mg RNase A for 1 hr at 37°C. DNA was isolated using the PureGene kit (Qiagen) and subjected to bisulfite modification using the MethylEasy Xceed kit (Human Genetic Signatures) according to the manufacturer's instructions.

PCR amplification and methylation sensitive high resolution melt analysis (MS-HRM) was performed in duplicate on the RotorGene™ Q (Qiagen). Primers were designed according to the principles outlined in [33] to control for PCR bias and are shown in Additional file 1: Table S1. PCR was performed using 2 ng of bisulphite modified template and standard PCR conditions, followed by one cycle of 1 min 30 sec at 72°C and an MS-HRM step from 70°C to 90°C rising by 0.1°C/sec. Bisulfite treated CpGenome™ Universal Methylated DNA (Chemicon, Millipore) and DNA from T47D were used as positive/methylated and negative/unmethylated controls, respectively. The methylation status of these controls was confirmed by direct sequencing of MS-HRM products, purified using the QiaQuick Gel Extraction Kit (Qiagen), performed by the Australian Genome Research Facility (AGRF, Brisbane, Australia). To create a range of methylated standards, these controls were mixed in 25, 50 and 75% methylated to unmethylated template ratios and were included in the analysis of each region.

### Sequenom MassARRAY DNA Methylation Analysis

Sequenom MassARRAY methylation analysis was performed as described previously [26]. The forward primer has a 10-mer tag (5-aggaagagag-3) and the reverse primer has a T7-promoter tag (5-cagtaatacgactcactatagggagaaggct-3) (Additional file 1: Table S1). Bisulfite treated CpGenome™ Universal Methylated DNA (Chemicon, Millipore) and Whole genome amplified DNA prepared as per instructions with the GenomePlex^® ^Complete Whole Genome Amplification kit (Sigma) were used as positive/methylated and negative/unmethylated controls, respectively. Triplicate PCR reactions were pooled and Shrimp Alkaline Phosphatase (Sequenom, San Diego) treatment performed followed by transcription and RNaseA Cleavage for the T-cleavage reaction. Purified samples were nanodispensed onto silicon chips preloaded with matrix (SpectroCHIPs; Sequenom, San Diego). Mass spectra were collected using a MassARRAY mass spectrometer (Bruker-Sequenom) and results analysed by the EpiTYPER software V 1.0. Methylation readings with overlapping signals and silent peaks were eliminated from the calculation.

### Quantitation of AR mRNA

To quantitate *AR *mRNA from cell lines, total RNA was extracted using Trizol (Invitrogen). cDNA was synthesised using 500 ng of RNA and Superscript First Strand Synthesis System III (Invitrogen), according to the manufacturer's instructions. β-actin was used to normalise mRNA concentration and primers are shown in Additional file 1: Table S1. Real-time PCR was performed in duplicate using the RotorGene™ Q (Qiagen) using 50 cycles of standard PCR conditions.

### Somatic mutation analysis

In breast cancer cell lines, a region spanning -659 to +280 with respect to the start of transcription (+1) were examined for somatic mutations. In FFPE tumours, the fragmented nature and limited availability of DNA meant that analysis was constrained to a smaller region (-165 to +133) and was examined in the 32 tumours for which IHC indicated negative AR expression. Primer sequences are shown in Additional file 1: Table S1. PCR was performed using KAPAHiFi DNA polymerase (KAPA Biosystems, Geneworks, Australia) and 50 ng of template using 30 amplification cycles as per the manufacturer's instructions. PCR reactions were purified using the QiaQuick Gel Extraction Kit (Qiagen) and sequencing performed by AGRF (Brisbane, Australia).

### Transcription factor binding site analysis

Bioinformatic analysis initially involved an analysis of UCSC Genome Browser (http://genome.ucsc.edu). Transcription factor binding sites were predicted by MOODS (MOtif Occurrence Detection Suite) [34]. MOODS uses the standard scoring model (log-odds against the background distribution) of PWMs. Scoring thresholds were specified by *P*-value less than or equal to 0.01. We tested T105A 5'UTR variant and WT sequences for overlap with the *TFBS *models in the *JASPAR *database [35]. TFBS logos were downloaded from the JASPAR database web server: http://jaspar.cgb.ki.se/.

### *AR *5'UTR reporter assays

The *AR *5'UTR T105A mutation was introduced into the wild-type *AR *5'UTR sequence (1116 bp, GenBank Accession No. NG009014.1) by site-directed mutagenesis using a two-step PCR procedure using the primers listed in Additional file 1: Table S1 and standard PCR conditions. The *AR *5'UTR wild-type and T105A mutant alone and together with the *AR *promoter were cloned into the *Hind*III/*Nco*I sites of pGL3-promoter and the *Kpn*I/*Nco*I sites of pGL3-basic (Promega), respectively. Constructs were confirmed by sequencing performed by AGRF (Brisbane, Australia).

MCF7 and T47D cells were transiently transfected with 800 ng pGL3+/- either the SV40 or the *AR *promoter, together with either the wild-type or mutant *AR *5'UTR sequence and 100 ng *Renilla *reporter in a 24-well plate, using Lipofectamine 2000 (Invitrogen). Forty-eight hours after the initial transfections, relative luciferase activities were determined using the Dual-Glo luciferase assay kit (Promega) and a DTX880 Multimode Detector (Beckman Coulter) according to the manufacturer's instructions. Statistical analysis was performed using unpaired, two-tailed t tests, with p values < 0.05 considered significant.

### miRNA analysis

Two algorithms, miRanda and TargetScan 5.1, were used to predict target sites for miRNA in the AR 3'UTR [36,37]. A total of 55 miRNA were predicted to target AR, 12 of which have conserved seed sequences. Only miR-124 was predicted by both algorithms. To quantitate the expression of miR-124, total RNA from transfected MCF7 and T47D cells was extracted using Trizol (Invitrogen) and the expression of miR-124 determined relative to RNU6B using the miScript PCR System according to the manufacturer's instructions (Qiagen).

### *AR *3'UTR reporter assays

The *AR *3'UTR (436 bp; GenBank Accession No. NG009014.1) was ligated downstream of the luciferase coding sequence in the pSG5 vector (Stratagene). The *mir*-124-1 stem-loop (84 bp; miRBase Accession No. MI0000443) ± 200 bp of flanking sequences was cloned into the *Kpn*I/*Xba*I sites of the pcDNA 3.1(+) expression vector (Invitrogen). MCF7 and T47D cells were transiently transfected with 400 ng pcDNA 3.1(+) constructs, 200 ng pSG5-luciferase constructs and 10 ng of *Renilla *reporter in a 24-well plate, using FuGene 6 (Roche). Forty-eight hours after the initial transfections, relative luciferase activities were determined using the Dual-Glo luciferase assay kit (Promega) and a DTX880 Multimode Detector (Beckman Coulter) according to the manufacturer's instructions. Statistical analysis was performed using unpaired, two-tailed t tests, with p values < 0.05 considered significant.

### Statistical analysis

Survival time was calculated from the date of tumour removal to the date of last follow-up or death attributable to breast cancer. Overall survival probabilities were estimated non-parametrically with the use of the Kaplan-Meier product limit method (GraphPad Prism version 5.0a) and statistical significance accepted as P ≤ 0.05.

## Competing interests

The authors declare that they have no competing interests.

## Authors' contributions

Kate M Peters^1^: Contributed to AR IHC analysis, developed AR MS-HRM assay, determined expression levels in Breast cancer cell-lines. Drafted manuscript. Stacey L Edwards^1^: Performed AR 5'UTR reporter assays. Shalima S Nair^2^: Performed AR Sequenom methylation assays on clinical samples and cell-lines. Juliet D French^1^: Peter J Bailey^1^: performed bioinformatics analysis of AR 5'UTR and promoter region. Kathryn Salkield^1^: Contributed to AR IHC analysis. Sandra Stein^3^: Managed breast tumour biobank, created tumour tissue array, optimized AR antibody IHC, performed AR, ER, PR and HER2 IHC. Sarah Wagner^3^: Contributed to creation of tumour tissue array and optimization of AR antibody IHC. Glenn D Francis^3^: Pathologist responsible to tumour bank, pathological review of tumour samples and analysis of ER, PR, HER2 and AR IHC results. Susan J Clark^2^: Contributed to project design, attracting research funding, interpreting results and review of manuscript. Melissa A Brown^1 ^Project leader. Contributed to project design, attracting research funding, interpreting results and reviewing, editing, submission and post-review revision of manuscript. All authors read and approved the final manuscript.

## Pre-publication history

The pre-publication history for this paper can be accessed here:

http://www.biomedcentral.com/1471-2407/12/132/prepub

## Supplementary Material

Additional file 1 **Table S1**. Oligonucleotide primers. Click here for file
