# Supplementary material for: Androgen receptor expression predicts breast cancer survival: the role of genetic and epigenetic events
Source: BMC Cancer. 2012 Apr 2;12:132. doi: 10.1186/1471-2407-12-132 (PMC3349557; doi:10.1186/1471-2407-12-132)
Supplement: Additional file 1 — Table S1. Oligonucleotide primers. [file 1471-2407-12-132-S1.DOC]

**Table 1** Patient and tumour characteristics*

| **Factor** | **AR negative**  **n=32 (%)** | **AR positive**  **n=41 (%)** |
| --- | --- | --- |
| Age (years) |  |  |
| ≤35 | 1 (3.0) | 1 (2.4) |
| ≥35 | 31 (97.0) | 40 (97.6) |
|  |  |  |
| Estrogen receptor |  |  |
| Negative | 23 (72.0) | 9 (22.5) |
| Positive | 9 (28.0) | 31 (77.5) |
|  |  |  |
| Progesterone receptor |  |  |
| Negative | 27 (87.0) | 16 (39.0) |
| Positive | 4 (13.0) | 25 (61.0) |
|  |  |  |
| HER2 |  |  |
| Negative | 18 (85.7) | 25 (75.8) |
| Positive | 3 (14.3) | 8 (24.2) |
|  |  |  |
| Triple negative^a^ |  |  |
| No | 6 (28.6) | 30 (90.9) |
| Yes | 15 (71.4) | 3 (9.1) |

^*^All patients were diagnosed with Stage III disease, as defined in the Materials and Methods section of the manuscript

^a^Triple negative breast cancer represents tumours displaying negative expression for estrogen receptor, progesterone receptor and HER2 by IHC. AR, androgen receptor; HER2, human epidermal growth factor receptor type 2.
